# SARCOPENIA AND POST-HOSPITAL OUTCOMES FOR OLDER PATIENTS IN AN EMERGENCY DEPARTMENT

**DOI:** 10.1093/geroni/igad104.3581

**Published:** 2023-12-21

**Authors:** Tai-Hung Ho, Hsien-Hao Huang

**Affiliations:** Taipei Veterans General Hospital, Taipei, Taipei, Taiwan (Republic of China); Taipei Veterans General Hospital, Taipei, Taipei, Taiwan (Republic of China)

## Abstract

**Introduction:**

Sarcopenia, an important condition in older adults, is associated with increased adverse outcomes, including falls, functional decline, frailty, and mortality. Sarcopenia is often not diagnosed in emergency department (ED) settings. Therefore, this study aimed to determine the prevalence, risk factors, and clinical outcomes of sarcopenia patients admitted to the ED.

**Methods:**

This observational study included patients aged ≥ 65 years admitted to the ED of Taipei Veterans General Hospital from September 2019 to November 2021. The patients were divided into two groups according to the definition of the Asian Working Group for Sarcopenia: sarcopenia and non-sarcopenia.

**Results:**

Among 867 enrolled, 447 (51.6%) were categorized into non-sarcopenia and 420 (48.4%) into sarcopenia groups. Sarcopenia patients were significantly older, shorter, weighed less, and had significantly lower values of body mass index, skeletal muscle mass, skeletal muscle index, and handgrip strength. The sarcopenia group had significantly fewer daily living and instrumental activities. The independent risk factors for sarcopenia were age (odds ratio [OR] 1.07, P < 0.001), malnutrition (OR 3.83, P < 0.001), and frailty (OR 1.91, P = 0.001). Sarcopenia patients had significantly higher admission (66.7% vs. 59.1%, P = 0.021) and 1-month mortality rates (P = 0.002).

**Conclusion:**

Sarcopenia patients are at risk of functional decline, hospital admission, and 1-month mortality. The independent risk factors for sarcopenia include age, malnutrition, and frailty. The recognition of sarcopenia as a medical condition in an ED may prompt targeted nutritional interventions, tailored discharge plans, and referrals to reduce disability, admission, and mortality.

